# Urokinase plasminogen activator (uPA) and plasminogen activator inhibitor type-1 (PAI-1) in breast cancer - correlation with traditional prognostic factors

**DOI:** 10.2478/raon-2014-0049

**Published:** 2015-11-27

**Authors:** Maja Lampelj, Darja Arko, Nina Cas-Sikosek, Rajko Kavalar, Maja Ravnik, Barbara Jezersek-Novakovic, Sarah Dobnik, Nina Fokter Dovnik, Iztok Takac

**Affiliations:** 1 Department of Gynaecologic and Breast Oncology, Division of Gynaecology and Perinatology, University Medical Centre Maribor, Maribor, Slovenia; 2 Division of Pathology, University Medical Centre Maribor, Maribor, Slovenia; 3 Division of Medical Oncology, Institute of Oncology Ljubljana, Ljubljana, Slovenia; 4 Faculty of Medicine University of Maribor, Maribor, Slovenia

**Keywords:** urokinase plasminogen activator, plasminogen activator inhibitor, breast cancer, prognostic factor

## Abstract

**Background:**

Urokinase plasminogen activator (uPA) and plasminogen activator inhibitor type-1 (PAI-1) play a key role in tumour invasion and metastasis. High levels of both proteolytic enzymes are associated with poor prognosis in breast cancer patients. The purpose of this study was to evaluate the correlation between traditional prognostic factors and uPA and PAI-1 expression in primary tumour of breast cancer patients.

**Patients and methods.:**

606 primary breast cancer patients were enrolled in the prospective study in the Department of gynaecological oncology and breast oncology at the University Medical Centre Maribor between the years 2004 and 2010. We evaluated the traditional prognostic factors (age, menopausal status, tumour size, pathohistological type, histologic grade, lymph node status, lymphovascular invasion and hormone receptor status), together with uPA and PAI-1. We used Spearman’s rank correlation, Mann Whitney U test and χ^2^ test for statistical analysis.

**Results:**

Our findings indicate a positive correlation between uPA and tumour size (p < 0.001), grade (p < 0.001), histological type (p < 0.001), lymphovascular invasion (p = 0.01) and a negative correlation between uPA and hormone receptor status (p < 0.001). They also indicate a positive correlation between PAI-1 and tumour size (p = 0.004), grade (p < 0.001), pathohistological type (p < 0.001) and negative correlation between PAI-1 and hormone receptor status (p = 0.002).

**Conclusions:**

Our study showed a relationship between uPA and PAI-1 and traditional prognostic factors. Their role as prognostic and predictive factors remains to be further evaluated.

## Introduction

Urokinase plasminogen activator system (uPAS) consists of urokinase plasminogen activator (uPA), tissue plasminogen activator (tPA), urokinase plasminogen activator receptor (uPAR), and plasminogen activator inhibitor type-1 (PAI-1) and type-2 (PAI-2). Proteolytic enzyme uPA converts the pro-enzyme plasminogen into proteolytically active form (plasmin), which takes part in physiological and pathophysiological processes on the basal membrane and inside the extracellular matrix, which are important for tumour growth and its metastases.^1^ Plasminogen activator inhibitor type-1, which functions as a natural inhibitor of uPA, is the most important factor among fibrinolytic inhibitors for the development of vascular diseases and cancer. uPA and PAI-1 do not only have proteolytic characteristics, but also have impact on the fundamental cellular processes, such as chemotaxis, migration, invasion, adhesion, proliferation and angiogenesis.^2^–^6^ uPA and PAI-1, as part of the fibrinolytic system, are the first factors with a confirmed clinical role in breast cancer (level of evidence I).^7^,^8^ According to the conclusions of the meta-analysis by Look *et al*.^7^, uPA and PAI-1 are in addition to axillary lymph node involvement the most important independent prognostic factors. uPA and PAI-1 are supposed to be useful in deciding upon adjuvant systemic therapy in women with low-risk primary breast cancer.^7^ Use of PAI-1 for therapeutic purposes has shown promising results on tumour models; however, the results are yet to be confirmed.^9^ The increase of PAI-1 could represent a response to the increased proteolytic activity caused by uPA inside the tumour. It is also possible that PAI-1 has a direct effect on the development of the disease.^10^

Prognostic and predictive factors are clinically important for planning the treatment of breast cancer, which improves disease-free survival, overall survival and quality of life. Prognostic factors predict the course of the disease independently of treatment and are connected with disease-free survival and overall survival. Tumour size, axillary lymph node involvement, pathohistological tumour type, malignancy grade and lymphovascular invasion are prognostic factors in the case of breast cancer. To assess patients with a high risk of recurrence, traditional prognostic factors do not suffice. Therefore, numerous studies are being conducted to discover better factors. uPA in PAI-1 are related to the course of breast cancer as statistically important independent prognostic factors.^11^–^15^ Numerous studies have shown that patients with low concentrations of uPA and PAI-1 have better survival than patients with high concentrations.^16^–^17^ The prognostic roles of DNA ploidy and S-phase fraction are not clearly defined yet.^18^

Predictive factors are biological markers by means of which it is possible to predict response to a certain type of treatment. Status of the hormone receptors, which predicts the response to hormonal therapy, and human epidermal growth factor receptor 2 (HER2) expression, which predicts the response to anti-HER2 therapy in patients with HER2-positive breast cancer, were confirmed to be reliable predictive factors in breast cancer. High level of evidence supports the predictive significance of uPA and PAI-1, which are the subject of many studies.^19^ Protein over-expression and/or amplification of the HER2 gene are found in around 20% of all breast cancer patients. Pre-clinical studies show that HER2 accelerates cellular adhesion and migration and therefore plays a key role in tumour cell invasion.^20^–^23^ Certain clinical studies indicate that in some cancer types HER2 stimulates the invasion of tumour cells with the effect on the accelerated release of proteolytic enzyme uPA and its inhibitor (PAI-1)^24^–^27^, whereas other studies did not confirm this assumption.^28^,^29^ The international coordinated guidelines, adopted at the conference in St. Gallen in 2007, require knowledge of factors such as tumour size, malignancy grade, age, axillary node involvement, status of hormone receptors and HER2 expression as the basis for choosing adjuvant therapy.^30^

Despite excellent evidence about the prognostic value of uPA and PAI-1, determination of these markers is not yet routinely used for planning adjuvant treatment. It is not completely clear if routine determination of uPA and PAI-1 would add important new information as opposed to simply confirming what can already be deduced from the traditional prognostic factors.

The aim of this study was to evaluate the correlation between uPA and PAI-1 and traditional prognostic factors in primary breast cancer. Statistically significant correlation between uPA and PAI-1 and traditional prognostic factors was expected. HER2 expression and its correlation with the traditional prognostic factors were also included.

## Patients and methods

### Patients

Six hundred and six patients with primary breast cancer, treated at the Department of Gynaecologic Oncology and Breast Oncology of the Division of Gynaecology and Perinatology, University Medical Centre Maribor, between the years 2004 and 2010 were included in this prospective study. The study was conducted in accordance with good clinical practice and all applicable regulatory requirements, including Declaration of Helsinki. The study was approved by the institutional review board and registered at Slovenian Research Agency under the clinical trial number P3-0321. All patients had pathohistologically confirmed invasive breast cancer. None of the patients had clinically or radiologically registered metastatic disease at the beginning of primary treatment. The characteristics of patients and tumours are presented in Table 1. Traditional prognostic factors such as menopausal status, pathological tumour size, pathohistological tumour type, malignancy grade, axillary lymph node involvement and lymphovascular invasion were assessed by means of clinical examination and pathohistological examination of tumour tissue. Tumours were classified according to the UICC-WHO criteria and malignancy grade according to Scarff-Bloom-Richardson (SBR) classification, modified by Elston.^31^ Lymphovascular invasion was evaluated as positive if tumour cell emboli were present in the vascular space lined by endothelium. Hormone receptors were evaluated by means of immunohistochemical staining of paraffin-embedded tumour tissue sections. Tumours in which at least 1% of tumour cells expressed oestrogen (ER) and/or progesterone (PR) receptors were marked as hormone receptor positive.

The study group was a cohort of women with breast cancer primarily treated at our institution during a period of seven years. During this time, recommendations for determination of some histological parameters have changed. Progesterone receptors and HER2 were not routinely determined in all patients throughout the study period and for some early cases of HER2 determination in situ hybridization was not performed in cases that were immunohistochemically marked as 2+. Besides, tumour grade was not reported for lobular histological subtypes in the past. Unfortunately, all this has lead to a high rate of missing data in these fields.

All patients were radically locally treated with a modified radical mastectomy or conservative operation (tumorectomy, quadrantectomy) and postoperative radiation. They further received adjuvant systemic therapy (chemotherapy and/or hormone therapy). Most patients with positive axillary lymph nodes and patients with a high risk and negative lymph nodes received adjuvant chemotherapy. All patients with positive hormone receptors received adjuvant hormone therapy.

### Laboratory measurements of uPA, PAI-1 and HER2

After histological examination of tissue sections, the tumour tissue obtained by surgery was stored for further analysis in liquid nitrogen. Samples of frozen tumour tissue were then pulverized with a Micro-dismembrator, dissolved in buffer (pH 5.5) composed of 0.02 M Tris-HCl, 0.125 M NaCl and 2% Triton X-100 and after 3 hours of stirring at 4°C centrifuged at 100,000 x g for 30 minutes. Protein content was measured with the bicinchoninic acid (BCA) method (Pierce, Rockford, IL). Antigens uPA and PAI-1 were quantified with standardized immunometric method using ELISA sets (American Diagnostica, Greenwich, CT, USA). Values of uPA and PAI-1 were expressed in ng/mg of proteins.

Based on the assesed intensity of membrane reaction due to the overexpression of HER onco-protein, the tumour tissue was categorized into one out of three groups: negative (0, 1+), equivocal (2+) and positive (3+). The immunohistochemically HER2 3+ result indicates positive HER2 status of the tumours. In all cases of the equivocal HER2 2+ results, the tumour tissue was retested with the fluorescence in situ hybridization (FISH) method using PathVysion^TM^ HER-2 PROBE KIT, in order to determine the amplification of HER2 proto-oncogene. All breast cancer patients with HER2 positive tumour status were treated with the monoclonal antibody trastuzumab.

### Statistical analysis

Spearman Rank Correlation was used to test the relation between continuous variables. We used the non-parametric Mann-Whitney U test to compare continuous and categorical variables and chi-square test for the comparison of categorical variables. Continuous variables uPA and PAI-1 were converted into binary variables to divide the patients into those with high risk and those with low risk by using limit values of 3 ng uPA / mg of proteins and 14 ng PAI-1 / mg of proteins.^32^ Statistical analysis was performed by means of the SPSS 17.0 program. The value of p < 0.05 was considered statistically significant. uPA and PAI-1 values are shown in Table 2.

## Results

606 patients with primary breast cancer were included in the prospective study. Mean age of patients was 60.1 ± 12.7 years. The youngest patient was 22 years old, and the oldest 95 years old.

### Correlation between uPA and PAI-1 values

**FIGURE 1.**
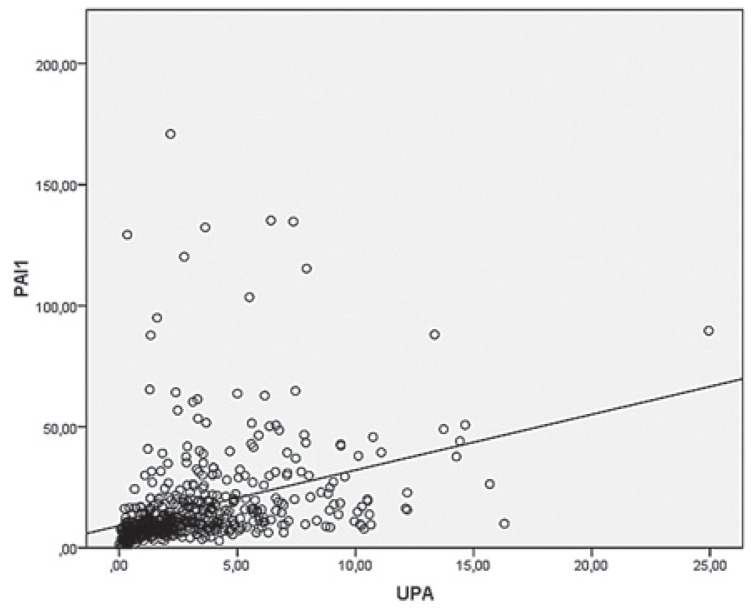
Correlation between urokinase plasminogen activator (uPA) and plasminogen activator inhibitor type-1 (PAI-1) values.

A strongly positive statistically significant correlation was established between uPA and PAI-1 values (r_s_ = 0.576, p < 0.001). The relation between uPA and PAI-1 values is shown in Figure 1.

### Correlation between uPA and PAI-1 and traditional prognostic factors

**FIGURE 2.**
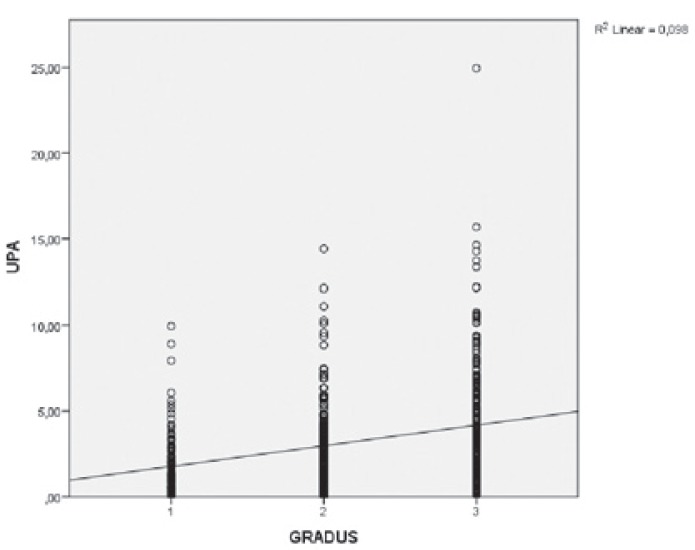
Correlation between urokinase plasminogen activator (uPA) and malignancy grade. Similar correlations were found for uPA and tumour size, pathohistological tumour type and lymphovascular invasion. Negative correlation was found between uPA and hormone and oestrogen receptor status. Analogous correlations between and plasminogen activator inhibitor type-1 (PAI-1) and all these factors except lymphovascular invasion were also determined.

Statistically significant correlation between uPA and PAI-1 and most of the traditional prognostic factors was established. They were related with tumour size, pathohistological tumour type, malignancy grade, status of oestrogen (ER) and hormone receptors. The relation between uPA and malignancy grade is shown in Figure 2.

No statistically significant correlation between uPA and PAI-1 and age, menopausal status, progesterone receptors (PR) and axillary lymph node involvement was found. Interestingly, only uPA’s correlation with lymphovascular invasion was statistically significant. The correlation between uPA, PAI-1 and traditional prognostic factors is shown in Table 3.

### Correlation between tumour HER2 positive status and traditional prognostic factors

HER2 tumour status was statistically related to all the traditional prognostic factors, except to axillary lymph node status (Table 3).

### Patients with a high risk due to high uPA and PAI-1 values and tumour HER2 positive status

High uPA levels were present in 223 patients (41%) and high PAI-1 levels in 195 (36%) patients, as shown in Table 2. High levels of one or both proteolytic enzymes, uPA and PAI-1, were identified in 288 (53%) patients. HER2 overexpression was identified in 127 (25%) patients.

Among 183 patients with high uPA, 50 patients (27%) had HER2-positive tumours. In the group of 263 patients with low uPA, 60 patients (23%) had HER2-positive tumours. Out of 157 patients with high PAI-1, 46 patients (29%) had HER2-positive tumours. Among 289 patients with low PAI-1, 64 patients (22%) had HER2-positive tumours. In the group of 238 patients with high values of one or both proteolytic enzymes, uPA and PAI-1, 65 patients (27%) had HER2-positive tumours and out of the 214 patients with low values of uPA and PAI-1 47 patients (22%) had HER2-positive tumours.

## Discussion

Prognostic and predictive factors play a key role in the proper treatment of breast cancer patients. Knowledge about these factors enables the patients with aggressive malignant tumours the possibility of adjuvant systemic therapy along with better survival and spares the patients with less aggressive malignant tumours unnecessary systemic treatment with numerous side effects while having the same chance of recovery. Establishing prognostic factors that are independent of treatment became very challenging in modern medicine, as most patients with breast cancer receive adjuvant systemic therapy after the primary treatment. It would be unethical to discontinue the adjuvant systemic therapy for research purposes regarding the characteristics of the disease.

The purpose of the study was to evaluate the relation between uPA and PAI-1 and traditional prognostic factors in primary breast cancer. HER2 overexpression as a prognostic and predictive factor and its correlation with traditional prognostic factors was also included in the study.

The sample size (606 patients) in our study is comparable with the reports by other authors.^33^–^39^ The study provides exact data on the characteristics of patients with primary breast cancer and their tumours. More than a third of patients (78%) were aged 50 or more and 73% of patients were postmenopausal. We determined that HER2 over-expression is more frequently present in younger and premenopausal patients. The same correlation was not established with uPA and PAI-1. Similarly, Look *et al*.^7^ found no significant relationship between uPA and age or menopausal status. However, they reported a correlation between PAI-1 and age and higher PAI-1 in postmenopausal women. They nevertheless considered these relationships not to be clinically meaningful.

Axillary lymph node involvement, tumour size, pathohistological tumour type, malignancy grade and lymphovascular invasion are the most important prognostic factors of the clinical course of breast cancer. In numerous studies these tumour characteristics were proved as independent prognostic factors since the disease recurred more often and affected the survival in patients with affected axillary lymph nodes, larger tumours, invasive ductal carcinoma, higher malignancy grade and lymphovascular invasion.

Numerous previous studies have shown that high levels of uPA and PAI-1 in primary tumour tissue negatively affect the outcome of breast cancer. uPA enables the development of metastases through proteolytic degradation of the extracellular matrix. Furthermore, PAI-1 also has an important role in invasion and metastasis, because it does not act only as inhibitor of uPA in plasminogen activator system but also affects most basic cell processes, such as adhesion, migration, invasion, proliferation and apoptosis of normal and malignant cells. Eljuga *et al.*^17^ even showed that PAI-1 determined immunohistochemically in tumour cells as opposed to the less available ELISA testing may carry important prognostic information in node-negative breast cancer patients.

A strongly positive correlation between both proteolytic enzymes, uPA and PAI-1, was established, which is in line with the findings of other studies.^7^,^28^,^29^,^34^,^36^ Despite this correlation, it is clinically important to determine both factors. Establishing both values allows differentiation between groups with high risk (with a high level of one or both factors) and making a decision on the proper individual adjuvant therapy.^40^

Size of the primary tumour is a known prognostic factor for the course of breast cancer. Patients with primary tumours equal to or larger than 2 cm, with a 53% share in our study, had more often high uPA and PAI-1 levels. A similar share of patients with large tumours (56%) was reported by Look *et al.*^7^ In larger tumours HER2 overexpression was also more frequently present.

Axillary lymph node involvement is related to tumour size, as larger tumours more often develop regional lymph node metastases. Interestingly though, high uPA and PAI-1 values in our patients were not related to axillary lymph node involvement. The reason may lie in the role of uPA and PAI-1 as independent prognostic and predictive factors in patients with primary breast cancer without axillary lymph node involvement. uPA was proved to be a stronger prognostic factor than tumour size, axillary lymph node involvement and oestrogen receptor status. Furthermore, it was also proved to be the strongest predictive factor of disease-free survival and overall survival in patients with primary breast cancer and no axillary lymph node involvement in numerous studies.^7^,^8^,^11^,^15^,^41^,^42^ In our study, 154 patients (40%) had a high uPA level and no axillary lymph node involvement. High PAI-1 levels were present in 136 patients (36%). High levels of both, uPA and PAI-1, were present in 88 patients (21%) with no axillary lymph node involvement. Individually or both, the uPA and PAI-1 levels were high in 202 patients (54%). De Cremoux *et al.*^42^ established high one or both levels of uPA and PAI-1 in 56% of patients with no axillary lymph node involvement, which is comparable to our results. Neither was axillary lymph node involvement related to the HER2 overexpression.

Malignancy grade of primary tumour is a confirmed prognostic factor in breast cancer. We have proved that patients with high levels of uPA and PAI-1 and HER2 overexpression more frequently have high-grade tumours. Poorly differentiated tumours (G2 and G3) were present in 74% of patients. Evaluating the malignancy grade by means of studying the histological structure and cytological characteristics of malignant cells is a subjective method. Sotiriou *et al.*^43^ found that it was necessary in patients with grade 2 tumours (that is 30–60% of all tumours) to determine the genomic grade index of the tumour. By doing so, the patients would be divided into those with a high and low risk for recurrence. G2 tumours were present in 35% of patients in our study.

We found that high levels of uPA and PAI-1 and HER2 overexpression are more often present in invasive ductal carcinoma than in lobular and other cancer types, which is in line with their different clinical outcomes. Invasive ductal type of breast cancer was present in 82% of our patients. Descotes *et al.*^39^ discovered 84% of invasive ductal breast cancer type.

Oestrogen and progesterone receptor status is an important predictive factor of the response to hormone therapy. Patients with positive ER and PR in the tumour have better survival than patients with positive only one type of hormone receptors. Patients with hormone receptor negative tumours have the worst survival rate. ER are mostly predictive factors of the response to hormone therapy, whereas PR are prognostic factors of the disease course, therefore it is important to measure both in the primary breast cancer tissue. In our study, high levels of uPA and PAI-1 had a negative correlation with ER, but no correlation with PR. HER2 overexpression had a negative correlation with both types of hormone receptors. We counted 79% of patients with positive ER. Other authors report a similar share of ER-positive tumours.^38^,^39^ PR-positive tumours were present in only 56% of our patients. The cause for the lack of correlation between uPA and PAI-1 with PR may lie in the missing PR values.

Lymphovascular invasion is an important morphological prognostic factor. Breast cancer develops metastases into regional and distant lymph nodes by lymphatic dissemination, and metastases into other parenchymal organs by hematogenous spread. Lymphovascular invasion was more frequently present in the tumour in patients with high uPA levels. There was no such correlation with PAI-1. Čufer *et al.*^36^ did not find any correlation between uPA and PAI-1 and lymphovascular invasion. In cases of lymphovascular invasion, HER2 overexpression was more often present.

High levels of one or both proteolytic enzymes were found in 53% of our patients and HER2 over-expression was present in 25%. A slightly higher percentage of HER2 overexpressing tumours was found in patients with high uPA, PAI-1 or both. A positive relationship between HER2 and proteolytic enzymes has been reported for other types of cancer^24^,^25^ but it has not been confirmed in breast cancer.^28^,^29^ This is the subject of our further research.

The results of our study on the correlation between uPA and PAI-1 and classic prognostic factors in primary breast cancer are concordant with the results of a meta-analysis of 8377 patients on the prognostic effect of uPA and PAI-1 in primary breast cancer.^7^ Correlation between uPA and PAI-1 and tumour size, pathological tumour type and hormone receptors and between uPA and axillary lymph node involvement are in line.

Further research of the characteristics of patients with primary breast cancer and their tumours as well as the definition of the role of proteolytic enzymes uPA and PAI-1 as prognostic and predictive factors in breast cancer is required.

## Conclusions

Urokinase plasminogen activator (uPA) and plasminogen activator inhibitor 1 (PAI-1) play a key role in invasion and metastases of malignant tumours. High levels of both proteolytic enzymes are related to poor prognosis in patients with breast cancer. By conducting this study we established that primary breast cancer patients with high values of uPA and PAI-1 usually have tumours that are larger, higher malignancy grade, invasive ductal pathohistological type and hormone independent. In cases of higher uPA lymphovascular invasion is more often present. We also established that HER2 overexpressing tumours occur more often in younger, premenopausal patients, are usually larger, hormone independent, of higher malignancy grade and invasive ductal histology, and they often show lymphovascular invasion.

Despite these significant correlations, it seems that uPA and PAI-1 values may help to additionally stratify especially node-negative breast cancer patients into different prognostic subgroups. In order to form a solid recommendation for or against routinely performing uPA/PAI-1 testing in breast cancer patients, further research about the prognostic and predictive impact of these factors in patients with primary breast cancer is required. The role of uPA and PAI-1 in survival of node-negative breast cancer patients is the subject of our ongoing research.

## Figures and Tables

**TABLE 1.** Characteristics of primary breast cancer patients and tumours (n = 606)

**Characteristics**	**Number of patients**	**Percentage (%)**
**Age**		
< 50 years	136	22
≥ 50 years	470	78
**Menopausal status**		
Premenopausal	162	27
Postmenopausal	444	73
**Pathological tumour size**		
< 2 cm	282	46
≥ 2 cm	319	53
Unknown	5	1
**Pathohistological classification of tumours**		
Invasive ductal	496	82
Invasive lobular	45	7
Other invasive	61	10
Unknown	4	1
**Malignancy grade**		
G1	126	21
G2	212	35
G3	235	39
Unknown	33	5
**Axillary lymph node involvement**		
Negative	333	55
Positive	243	40
Unknown	30	5
**Oestrogen receptors**		
Negative	119	20
Positive	478	79
Unknown	9	1
**Progesterone receptors**		
Negative	219	36
Positive	337	56
Unknown	50	8
**Hormone receptors**		
Negative	101	17
Positive	492	81
Unknown	13	2
**Lymphovascular invasion**		
Yes	103	17
No	481	79
Unknown	22	4
**HER2**		
Negative	127	21
Positive	373	62
Unknown	106	17

G = grade; HER2 = human epidermal growth factor receptor 2

**TABLE 2.** Levels of urokinase plasminogen activator (uPA) and plasminogen activator inhibitor type-1 (PAI-1) in tumour tissue of primary breast cancer patients

	Range^*^ (min–max)	Median value^*^ (25 / 75 percentile)	Limit values^*^	**Number of patients**	**Percentage (%)**
uPA	0–24.95	2.34 (1.08 / 4.20)	< 3 ≥ 3	319 223	59 41
PAI-1	0–170.92	10.6 (6.93 / 18.27)	< 14 ≥ 14	347 195	64 36

* ng / mg of proteins

**TABLE 3.** Correlations between urokinase plasminogen activator (uPA), plasminogen activator inhibitor type-1 (PAI-1), human epidermal growth factor receptor 2 (HER2) and traditional prognostic factors

**Variables**	Number of patients^*^	**uPA**	**PAI-1**	**HER2**
**Age**				
< 50 years	136	p = 0.700	p = 0.402	**p = 0.017**
≥ 50 years	470			
**Menopausal status**				
Premenopausal	162	p = 0.896	p = 0.218	**p = 0.008**
Postmenopausal	444			
**Pathological tumour size**				
< 2cm	282	**p < 0.001**	**p = 0.004**	**p = 0.005**
≥ 2cm	319			
**Pathological tumour type**				
Ductal invasive	541	**p < 0.001**	**p < 0.001**	**p = 0.021**
Other invasive	61			
**Malignancy grade**				
G1 + G2	338	**p < 0.001**	**p < 0.001**	**p < 0.001**
G3	235			
**Axillary lymph node involvement**				
Negative	333	p = 0.052	p = 0.171	p = 0.385
Positive	243			
**Oestrogen receptors**				
Negative	119	**p < 0.001**	**p < 0.001**	**p < 0.001**
Positive	478			
**Progesterone receptors**				
Negative	219	p = 0.162	p = 0.960	**p = 0.003**
Positive	337			
**Hormone receptors**				
Negative	101	**p < 0.001**	**p = 0.002**	**p < 0.001**
Positive	492			
**Lymphovascular invasion (LVI)**				
Yes	103	**p = 0.010**	p = 0.292	**p = 0.006**
No	481			

* Due to missing values the number of patients is not always 606; G = grade
